# Declining resilience of ecosystem functions under biodiversity loss

**DOI:** 10.1038/ncomms10122

**Published:** 2015-12-08

**Authors:** Tom H. Oliver, Nick J. B. Isaac, Tom A. August, Ben A. Woodcock, David B. Roy, James M. Bullock

**Affiliations:** 1 NERC Centre for Ecology and Hydrology, Maclean Building, Benson Lane, Crowmarsh Gifford, Wallingford, Oxfordshire OX10 8BB, UK; 2 School of Biological Sciences, University of Reading, Whiteknights, PO Box 217, Reading, Berkshire RG6 6AH, UK

## Abstract

The composition of species communities is changing rapidly through drivers such as habitat loss and climate change, with potentially serious consequences for the resilience of ecosystem functions on which humans depend. To assess such changes in resilience, we analyse trends in the frequency of species in Great Britain that provide key ecosystem functions—specifically decomposition, carbon sequestration, pollination, pest control and cultural values. For 4,424 species over four decades, there have been significant net declines among animal species that provide pollination, pest control and cultural values. Groups providing decomposition and carbon sequestration remain relatively stable, as fewer species are in decline and these are offset by large numbers of new arrivals into Great Britain. While there is general concern about degradation of a wide range of ecosystem functions, our results suggest actions should focus on particular functions for which there is evidence of substantial erosion of their resilience.

## Abstract (web summary)

Global change may affect the resilience of ecosystem functions by altering community composition. Here, Oliver *et al.* show that in Great Britain since the 1970s there have been significant net declines among animal species that provide key ecosystem functions such as pollination and pest control.

## Graphical abstract

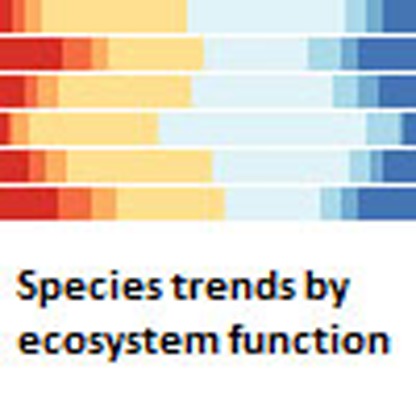

Biological species are essential for the provision of ecosystem services ranging from food production (including direct food provision and the underpinning functions of pollination, pest control and decomposition), climate regulation (carbon sequestration) to intrinsic cultural values^1^. More biodiverse systems, in particular those with higher species richness, have often been found to provide higher levels of ecosystem function under controlled experimental conditions^2^,^3^. Perhaps more importantly, and our focus here, is the additional role of biodiversity in maintaining ecosystem function flows in the longer term under environmental perturbations^3^,^4^, that is, promoting resilience in function provision^5^.

Although there is clear evidence of declines in biodiversity (taxonomic, phylogenetic and functional) at the global level^6^, the impact on the resilience of ecosystem functions on which humans depend is not known. Understanding which functions are more or less at risk is important for prioritizing conservation and restoration efforts. In theory, long-term trends in species occurrence can be linked to temporal change in the resilience of ecosystem functions, in order to identify large-scale patterns and help inform planning of national and international responses. However, progress has been hampered through: (a) a lack of data and robust methodology to calculate trends in the frequency of occurrence of functionally important species from opportunistic biological records (the most common source of ecological data for species) and (b) a lack of information on ‘effect' traits, which are attributes that determine the contributions of species' individuals to ecosystem function^7^.

To assess trends in the occurrence of species, data availability is often a limiting factor, with previous attempts being restricted to a subset of species groups for which standardized monitoring data are available—in Great Britain, this comprises a subset of mammals, birds, butterflies and macro-moths. In this study, for these four species groups with standardized monitoring schemes in place (395 species), we calculated trends in individual species' abundances over the last four decades. For an additional 4,029 species from 18 national recording schemes, we applied new analytical methods to calculate trends in frequency of occurrence from nonstandardized occurrence records, accounting for spatiotemporal patterns in recorder effort. We used binomial mixed effects models^8^ to estimate trends in frequency of occurrence across 1 km grid cells for each species in Great Britain between 1970 and 2009. This approach has been shown to be robust to spatiotemporal variation in recorder effort in a simulation study comparing different methods^8^. It produces trends in species' occurrence that reflect national and local abundance trends, where data are available for comparison. For each species' model, we tested the null hypothesis of no trend in occurrence over time, at three different thresholds of type 1 error: 0.05, 0.01 and 0.001.

Next, we grouped all species by the primary ecosystem functions that they underpin, namely: decomposition, carbon sequestration, pollination, pest control and cultural values. Our assumption is that changes in the national frequency of species in each functional group can provide an indication of trends in resilience of those functions. Our argument is that when more species are present in a functional group there is a ‘portfolio' effect whereby the overall abundance of individuals providing the function is more constant owing to a statistical averaging effect^4^,^9^, meaning levels of function provision are less likely to fall below some minimum acceptable threshold^5^,^10^. Furthermore, there is often negative spatial and/or temporal covariance (asynchrony) between species' population sizes, driven by differing responses to environmental change or competition^4^,^9^,^10^. These mechanisms lead to an ‘insurance' effect of biodiversity (also sometimes called ‘functional redundancy') whereby higher species richness within a functional group is more likely to maintain ecosystem function provision under environmental perturbations, that is, it leads to more resilient ecosystem functions.

Rather than compare absolute numbers of declining species across functional groups, we assessed the balance of increasing versus decreasing species using proportion tests and presented results as log ratios, so that our tests were not biased by differences in total species numbers or statistical power between functional groups arising from differences in the mean numbers of records per species. We repeated the tests at each threshold of type 1 error to assess sensitivity to the level of statistical significance for trends.

To put species into functional groups, we consulted taxon experts and reviewed published literature to classify which higher taxa are primary or secondary providers of pollination, pest control, decomposition, carbon sequestration and experiential cultural values. Although individual species vary in their functional contributions^11^,^12^, our reasoning for allocating ecosystem function provision at three broad levels (‘primary', ‘secondary' or ‘very limited/none') for higher taxa, as opposed to species-specific weightings, is twofold. First, functional contribution measurements are often context specific as the roles of species can change over space and time^4^,^10^,^13^. In different environments, species' relative frequencies vary, affecting their functional contributions^10^,^14^,^15^, while there are also changes in the per capita contributions of individual species to function provision^7^. For example, the per capita roles of natural enemies vary depending on which crop pest is dominant and which other natural enemies are present^13^,^16^. Therefore the limited ‘effect trait' data that do exist^17^,^18^,^19^ may not accurately predict ecosystem functions provided by individual species in new locations or time periods. A second reason for our species grouping is that environmental resource managers are tasked with ensuring the continued provision of ecosystem functions under changing environments, that is, their resilience^5^. This includes maintaining ecosystem functions in the face of the challenges posed by climate change, habitat degradation, invasive species and pollution. Because the dominant species providing ecosystem functions can switch under these perturbations, an assessment of the resilience of functions must consider all of the species that can potentially fulfil a function (that is, reflecting the ‘portfolio' or ‘insurance' effect of biodiversity^2^,^3^), rather than on the small subset of species that are currently functionally dominant^20^,^21^.

To evaluate the consequences of observed biodiversity change for the resilience of ecosystem functions, we explored the balance between increasing and decreasing species within each functional group. We also calculated the frequency of new species arriving in Great Britain since 1970, to assess the likelihood that disproportionate declines of species in a given functional group might be offset by new arrivals.

Many ecosystem functions are delivered at the local level (and, therefore, their resilience is determined by regional species pools), whereas our analysis reflects changes in the frequency of species in functional groups at the national level. However, there are strong reasons to believe that these national trends will also reflect changes in average regional species richness. Each trend in the frequency of occurrence of a species at the national level is derived from local changes in occupancy at the 1 km scale, and thus reflects average changes at this scale. Collating trends by functional group, there may be 1 km cells which are the exception and retain higher numbers of species (for example, protected areas with higher-quality habitats) and, equally, cells which lose species more rapidly (for example, intensive farmland). However, on balance, national trends in species richness should also be reflected by changes in average regional species richness, especially as the species richness at larger spatial scales forms the species pool for smaller scales.

There are likely to be other factors besides regional species richness which may also affect the resilience of ecosystem functions. For example, mechanisms at the intraspecific level such as genetic diversity and at the landscape scale such as habitat diversity and connectivity may also have a role in mediating ecosystem function resilience^5^. Notwithstanding these points, species richness (and the functional redundancy it confers) is seen as a key mechanism in promoting resilience of ecosystem functions^2^,^3^,^4^,^9^,^10^ and the national species trends from 22 higher taxonomic groups assembled here provide a key source of evidence to indicate trends in the resilience of ecosystem functions over the last four decades.

Our results show that the resilience of particular ecosystem functions, such as pollination, is being eroded more rapidly than others. This has implications for prioritizing ameliorative actions to limit the likelihood of deficits in the provision of ecosystem functions and the consequent negative impacts on human well-being that these would have.

## Results

### Trends in species across functional groups

Trends in the frequency of occurrence of 4,029 species from 1970 to 2009 were analysed using binomial mixed effects models (see Methods), and these were combined with data for an additional 395 species for which abundance trends were already available. In the Supplementary Information, we provide an example for butterflies showing how trends such as these estimated at the national level are also reflective of trends at the regional level (Supplementary Fig. 1).

**Figure 1:**
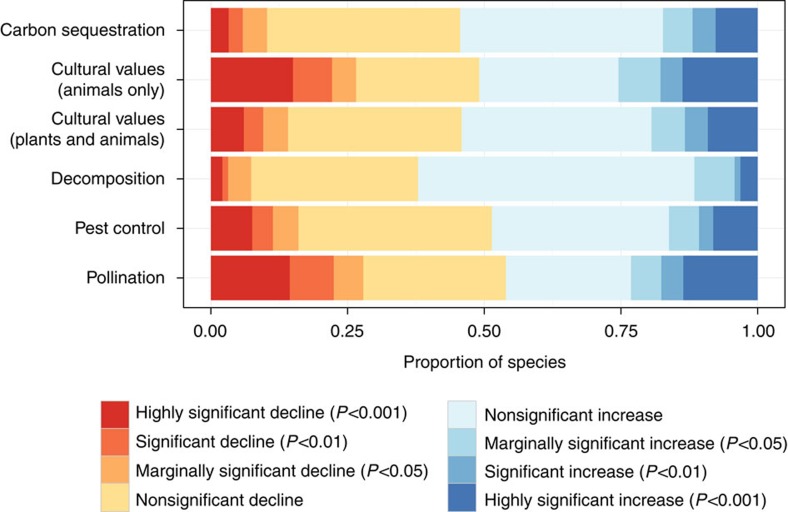
Trends in species grouped by ecosystem function. Shown are the proportion of species in different functional groupings showing significant changes in frequency of occurrence in Great Britain between 1970 and 2010. Total sample sizes for respective rows are as follows: *n*=2,276; 590; 2,615; 95; 1,447; 720.

**Figure 2:**
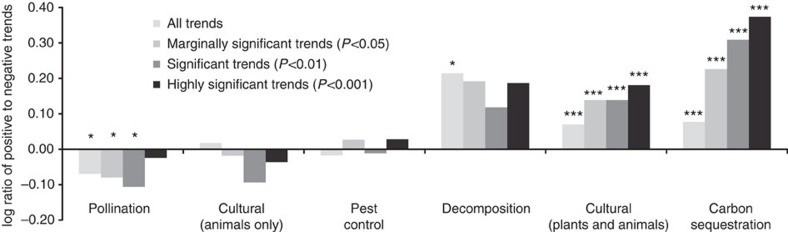
Net balance of species trends across ecosystem functions. Shown are the log ratio of numbers of increasing versus decreasing species in different functional groups. The different bars indicate different significance levels for individual species trends. A positive ratio indicates more species in a given functional group are increasing. Differences in the balance of increasing versus decreasing species is assessed using an exact binomial test for all trends or a proportion test for significant trends. Asterisks indicate significantly different proportions (*P<0.05; ***P<0.001).

Our overall analysis showed that species groups providing decomposition and carbon sequestration functions appear relatively robust. Only 7 and 10% of species (*n*=95 and 2,276) in these respective groups have shown statistically significant declines (assessed at *P*<0.05; proportion test), and there are a greater number of increasing species (12 and 17% of species have increased, respectively; Figs 1 and 2; Supplementary Table 2). However, groups providing pest control or pollination functions have shown greater declines of 16, and 27% of species, respectively (*n*=1,447 and 720 species). For pest control, these declines have been largely offset by increases in other species (17%), but this is less so for pollinating species, of which only 23% of species are increasing.

For cultural values, considering all species in this functional grouping, declines were more than offset by increases (14 versus 19%; *n*=2,615; Figs 1 and 2; Supplementary Table 2). However, considering only the animal species that provide cultural values, the decreases and increases were much more balanced (27 versus 25%, *n*=590).

### Comparison with newly arriving species

**Figure 3:**
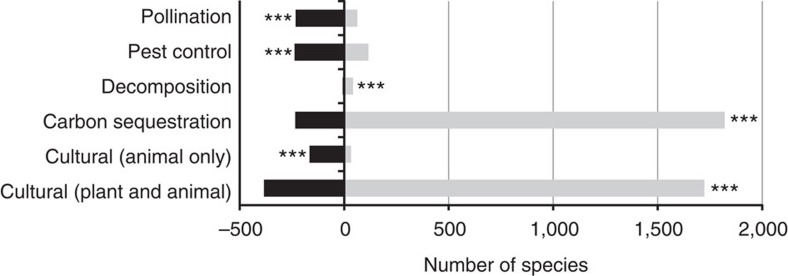
Balance of declining species versus new arrivals grouped by ecosystem function. Shown are numbers of species with declines in frequency of occurrence in Great Britain between 1970 and 2010 (at *P*<0.05; black bars) versus the number of new species arriving into Great Britain since 1970 (grey bars). Asterisks indicate significantly different proportions using an exact binomial test (***P<0.001).

These trends are in native species or those present within Great Britain before 1970. A number of species have arrived in Great Britain since 1970, principally through human introduction, and therefore analysis of resident biodiversity change only tells half the story. We found that a large number of species that can provide carbon sequestration, decomposition and cultural values (plant and animals combined) have arrived in Great Britain since 1970. These additions to national biodiversity, in combination with the large number of increasing native species, offer further potential to offset the relatively small numbers of declining species (Fig. 3). This suggests that the resilience of carbon sequestration and decomposition should be relatively robust despite wider biodiversity decline. In contrast, species groups providing pollination, pest control or animal-associated cultural values have had far fewer species arriving relative to the numbers that are in decline. Therefore, these ecosystem functions appear to be under particular threat. Results remain qualitatively similar when ‘secondary' function providers (groups considered to have only a minor contribution to a function) are also included, except that decomposers show a much greater proportion of species increasing in frequency of occurrence (Supplementary Figs 2–4; Supplementary Table 3).

### Exploring finer resolution functional classifications

One additional question that may be asked, which is relevant to informing management of ecosystem functions, is whether trends in the frequency of occurrence of all species in a broad functional group (for example, ‘pest control providers' or ‘pollinators') also reflect patterns in a subset of those species that are particularly important for a specific ‘sub-function' (for example, pest control in wheat or pollination of oilseed rape—two of the most widespread crops in Great Britain). To explore this, we analysed trends for the subsets of carabid beetles that provide pest control in wheat and of bees that pollinate oilseed rape^22^ and compared these with trends for all British carabids and bees, respectively (Supplementary Figs 5 and 6). In both the cases, we found no significant difference in the proportion of species showing positive or negative trends (at *P*<0.05; proportion test). This suggests that our results can be broadly indicative of a more refined classification of crop-visiting species within these broad functional groups.

## Discussion

Our results show significant variation in biodiversity trends between different broad functional groups. For those functional groups which have suffered net declines of species, this does not necessarily mean that there have been reductions in the provision of these functions; the species that declined may have had less dominant functional roles under recent environment conditions^20^. Alternatively, the ‘functional redundancy' effect of biodiversity may have buffered these losses, whereby declines in functionally important species are replaced by increases in others^4^,^5^,^9^,^10^. However, our results do provide evidence for erosion of the resilience of certain functions, increasing the risk of failure in their delivery under future environmental perturbations. Loss of species richness in functional groups means that there is a weaker ‘portfolio' effect (independent fluctuations of multiple species leading to a more stable ecosystem function provision^4^,^9^), as well as lower functional redundancy^4^,^5^,^9^,^10^. Therefore, the ‘insurance' capacity provided by biodiversity is weakened leading to higher risk of ecosystem function deficits. The extent of this risk is a function of both the relative number of functionally important species that are declining in combination with the magnitude and impact of future environmental perturbations. Some perturbations such as extreme weather events are predicted to continue to increase in the future^23^. We do not address changes in perturbation frequency here; our results inform on the extent of declines in functionally important species, but the insurance value provided by these species is likely to be even higher under the more frequent and higher magnitude perturbations expected in the future. Therefore, it is important to conserve biodiversity to maintain resilient ecosystem functions.

Our results show some declines in species across all functional groups (Fig. 1, Supplementary Table 2); but for carbon sequestration and decomposition, the relative proportion of declining species is small and more than offset by increasing species and new arrivals into Great Britain. Therefore, these functions are likely to remain relatively resilient. However, for pest control and pollination, the arrivals into Great Britain are not sufficient to offset declines, which suggests an erosion of resilience of these ecosystem functions. Recent work has shown that most crop pollination is carried out by just a few species that are common and not particularly threatened^20^,^21^. However, significantly different environmental conditions, such as those expected under climate change, can force species outside of their niches causing large declines^24^. In these cases, less abundant species may replace dominant functional roles, but only if sufficient species richness has been maintained to conserve this functional ‘redundancy'^21^. Therefore, maintaining biodiversity is essential to safeguard pollination of both crops and wildflower plants.

For the cultural values arising from biodiversity, it is not yet clear the extent to which plants and animals are complementary functional groups with respect to their impacts on wellbeing^25^. Nor is it known whether the cultural values of native versus non-native species differ for most people. Cultural values assessed across plants and animals combined appear to be resilient owing to limited declines in native species, but the animal-associated component has suffered greater proportional declines which are not offset by new arrivals.

In this study, we took the approach of categorizing ecosystem functions at broad taxonomic levels (Table 1). Notwithstanding the current lack of knowledge of the relative functional roles of many species, it is likely that the functional contributions of species are context specific and vary between locations and over time^4^,^10^,^13^. This is particularly likely to be the case where environmental conditions show large temporal or spatial variation, such as may occur under climate change^14^,^24^. Therefore, allowing for potential shifts in ecological dominance with environmental change, resource managers are best to consider the full suite of species which can fulfill a given functional role to assess the resilience of an ecosystem function^5^. Future work could investigate the ideal taxonomic resolution at which functional redundancy operates for different ecosystem functions. For example, Kleijn *et al.*^21^ show that only a relatively small subset of bee species are important for the pollination of European crops under current environmental conditions, although this primarily reflects species' relative abundances^20^, which could easily change in the future^21^. Here, we assessed trends in a much broader range of pollinating species, but we also repeated the analysis for the subset that are commonly found pollinating oilseed rape, the most common insect-pollinated crop in Great Britain. In this case, we did not find marked differences in the balance of declining versus increasing species between the two sets of functional categorization, although this is not always guaranteed to be the case. Of course, as well as the time frame over which resilience is assessed, the exact function of interest is also pertinent. An interest in the resilience of pollination of wildflowers and crops in general would lead to inclusion of a much broader range of pollinating species than focus on the resilience of pollination of a specific crop. Likewise, allowing for flexibility in exact crop variety or species (for example, for optimum crop choice varying over time owing to climate change or fluctuations in global markets), one may wish to consider a broader range of potential functional species.

The ecosystem functions we studied are associated positively with ecosystem services, such as crop production or climate regulation. We conducted a sensitivity analysis by excluding species that may have negative impacts in relation to societal needs (that is, they provide ‘disservices', for example, by acting as crop pests) and might negate or outweigh certain functions we considered (for example, excluding species that are pollinators in the adult stage but crop pests in the larval stage). However, we did not attempt to assess the resilience of such ecosystem disservices (that is, ‘unhelpful' resilience^26^). The reason for this is that ecosystem disservices are more likely to be a result of the actions of individual species, rather than suites of functionally similar species. Therefore, other metrics besides species richness and associated functional redundancy are more likely to be relevant in assessing the resilience of these disservices (for example, genetic diversity of pest species helping them to develop resistance to pesticides, or changes in landscape habitat structures helping disease vectors to spread^5^). Thus, it should be noted that some of the species here may have negative impacts in some contexts, and these may need to be managed on an individual basis.

With regards to the five ecosystem functions that we considered, our overall results suggest that widespread concerns that biodiversity declines will compromise ecosystem functions and the services they underpin^1^,^3^ are well founded, but our results suggest that certain functions are less resilient and at higher risk than others. Efforts to reverse losses in biodiversity and ecosystem services, such as ecological restoration, necessarily involve trade-offs with different actions benefiting different taxa and services^27^. Restoration actions can also take decades to become effective^28^. By indicating which ecosystem functions are most at risk, this study provides a possible approach to prioritizing ameliorative actions. However, continued research into species' functional roles and monitoring of their status, especially the development of monitoring schemes for less well-studied but functionally important groups, such as soil invertebrates and microorganisms, is critical for refining risk assessments and guiding sustainable environmental management.

## Methods

### Statistics of species' abundance and occurrence trends

Where standardized abundance data were available for taxonomic groups we used these (birds: http://www.bto.org/volunteer-surveys/bbs; butterflies: http://www.ukbms.org/; moths: http://www.rothamsted.ac.uk/insect-survey/LTTrapSites.html; mammals: http://jncc.defra.gov.uk/trackingmammals). For butterflies and moths, abundance trends and associated confidence scores were available from log-linear Poisson models^29^ fitted to data across all sites for the dates 1976–2012 (ref. 30) and 1968–2007, respectively^31^. For moths, these abundance data reflect a subset of all species in Great Britain. Therefore, we multiplied the number of new moth arrivals identified from occurrence data by the proportion of British moth species for which abundance trends were available to ensure a fair comparison. For birds, trends were derived from fitting a linear regression to annual combined indices from the Breeding Bird Survey and Common Bird Census Schemes between 1970 and 2009 (ref. 32). For mammals, trends were only available over a 25-year period up to 2007 for 21 species. Precise statistics, beyond qualitative indication of significance at *P*<0.05, are not published in the Tracking Mammals Partnership Update^33^, so any trends were conservatively allocated as marginally significant at 0.01<*P*<0.05. For a further 10-bat species, trends were only available from 10 years before 2007. Because of the short timeframe relative to the rest of our analysis (1970–2010), any significant 10-year trends were treated as having low confidence (*P*>0.05) over the entire timeframe.

For species groups without standardized abundance monitoring schemes, geo-referenced species occurrence records with sighting dates were obtained from 18 data sets from national recording schemes and societies in Great Britain. For each species, a binomial linear mixed-effects model was fitted to detection/non-detection data of species in selected 1 km cells across Great Britain, to assess directional changes over time (increase or decrease) in the probability of species occurrence per ‘site visit'. This probability of species occurrence relates to both the number of cells occupied (that is, the distribution extent of a species) and to the local abundance of species in the average cell (Supplementary Fig. 1). Across many species, for any given cell, these changes will lead to a net change in the number of function-providing species present and their abundances, with potential consequences for resilience of ecosystem functions^2^,^3^,^4^,^9^,^10^.

A ‘site visit' to each 1 km cell is defined as a unique combination of date, 1 km^2^ grid cell and taxonomic group (that is, those listed in Table 1). To reduce the variation in recorder effort, we restricted analyses to well-sampled grid squares with repeat visits by filtering data. This was done by first removing all visits where the total number of species recorded was less than the median for the taxonomic group in question. Second, we excluded all grid cells that had visits in fewer than 3 years between 1970 and 2009 (ref. 34). This determined the total sample size for statistical analysis of each species. A mixed-effects model, with binomial error structure, was then fitted to the detection/non-detection data of these 1 km cells with year as the covariate and 1 km grid cell as a random effect^34^,^35^. For each species' model, we tested the null hypothesis of no trend in occurrence over time, at three different thresholds of type 1 error: 0.05, 0.01 and 0.001 and collated results for all the species in each functional group. We assessed the balance of increasing versus decreasing species using proportion tests and presented results as log ratios, repeating tests at each threshold of type 1 error.

### Categorizing species groups by ecosystem function

For each of the five ecosystem functions assessed, our sample sizes were determined by the availability of species data. However, our analyses contained at least one or more of the broad species groups of key importance as assessed by the UKNEA^36^ (Supplementary Table 1). Notwithstanding this, microorganisms and fungi are also important for the provision of some functions such as decomposition, carbon sequestration and pest control, but lacked sufficient data for analysis^36^. Therefore, a caveat in our analysis is that trends in these unstudied groups could affect the performance of ecosystem functions. However, we do include a large component of the biodiversity underpinning these ecosystem functions, and the effects of other taxa such as microorganisms and fungi are likely to be either complementary or additive to the species we analyse.

In total, we analysed 4,424 species across 22 groups defined by national recording schemes. The taxonomic resolution of these varies (Table 1), but such grouping was preferable to deal with spatiotemporal variation in recorder effort (records on species are collated and validated within each recording scheme). In addition, species within a recording scheme often share the same functional roles (for example, all plants sequester carbon, all bees visit flowers and potentially carry pollen), although average functional contributions may differ and be context specific^11^,^13^,^37^. However, for pest control, strictly herbivorous or granivorous species clearly do not deliver this function and so were excluded from this group. The following literature resources were used to exclude six ladybird beetle species (Coleoptera: Coccinellidae)^38^,^39^,^40^,^41^,^42^,^43^, 21 grasshopper and cricket species (Orthoptera)^44^,^45^, four bird species (Aves)^46^ and nine mammal species (Mammalia). In addition, some species may provide ecosystem disservices, such as crop pests. These species may provide an ecosystem function such as pollination in the adult stage, but larval stages may be pests, with a net negative effect on ecosystem services of crop production. Although this study focuses on ecosystem functions rather than final services (for example, food production), we investigated the exclusion of temperate crop pest species (81 species identified from ref. 47), but found negligible differences in our overall results (Supplementary Fig. 7).

For each ecosystem function, a combination of expert consultation and published literature was used to classify species groups into primary or secondary providers of an ecosystem function (Table 1; note that groups can provide multiple functions). This process comprised the authors first allocating all species groups by function based on our own ecological knowledge, then contacting experts to review and suggest modifications to these groupings. A minimum of two experts per ecosystem function, identified on the basis of relevant career history, contributed to the process. To support and validate the evidence provided by expert opinion, a subsequent literature review was then undertaken to highlight relevant references for the final species groupings. Primary function providers are those groups where the majority of species contribute in an important way to delivering that function. Analyses were subsequently repeated including ‘secondary' function providers, which were groups whose species can potentially provide those ecosystem functions, but to a more limited extent. Where experts did not agree or there was only limited evidence for the functional role of a given species group, that group was classed as a secondary function provider.

Although the species occurrence records analysed in this study represent an unparalleled resource of millions of records (1,546,816 records analysed after strict controls for recorder effort), a number of species groups did not have sufficient data for analysis. For example, certain fly species (Diptera) may contribute to pollination functions, but sufficient records for fly families other than Syrphidae (hoverflies) were lacking. In many cases, this is due to the taxonomic intractability of certain taxa that have precluded the long-term collection of biological records. Nevertheless, for pollination and most of the other functions assessed in this study, we are confident that key species groups are represented in our analysis. Significant exceptions are earthworms (Clitellata: Oligochaeta) and microorganisms, which include highly important decomposers^48^, but lack sufficient data to be included in the analysis. Therefore, further monitoring might prioritize such important groups to refine future analyses. A full list of species included in the analysis, the group to which they belong and their category of ecosystem function contribution can be found in Supplementary Data 1. A summary of the species groups included in each function follows below.

*Pollination*. Primary pollinating species groups were identified as those which repeatedly visit flowers of the same species and have body structures and behaviours which could lead to a reasonable likelihood of pollen transfer. Bee species (Hymenoptera: Apidae) are regarded as highly effective pollinators^49^,^50^, but other groups such as hoverflies (Hymenoptera: Syrphidae), moths and butterflies (Lepidoptera) also provide important outcrossing pollination functions^50^. Secondary pollinating species groups included two beetle groups: soldier beetles and glow-worms (Coleoptera: Cantharidae, Drilidae and Lampyridae) and cerambycid beetles (Coleoptera: Cerambycidae) that often interact with flowers but are not thought to transfer much pollen owing to their relatively smooth bodies when compared with bees^49^. Wasps (Hymenoptera: Apoidea, Chrysidoidea and Vespoidea) were also included as secondary pollinators reflecting their frequent visitations to flowering plants^51^.

*Pest control*. Species groups providing primary pest control functions were those predators that are likely to act as natural enemies of crop pests. This includes carabid beetles (Coleoptera: Carabidae)^52^,^53^, ladybird beetles^52^,^54^,^55^, spiders (Araneae)^52^,^54^,^56^, centipedes^52^, wasps (for example, Ichneumonidae and Brachonidae)^57^, dragonflies and damselflies (Odonata)^58^, harvestmen (Opiliones)^56^,^59^, hoverflies 54,60, soldier beetles and glow-worms^56^, ants (Hymenoptera: Formicidae)^61^; although some ant species can also protect aphid crop pests^62^, birds^63^ and mammals (Mammalia)^64^,^65^. Omnivorous crickets (Orthoptera) and earwigs (Dermaptera)^44^,^45^ may act as predators and were included as providing secondary pest control.

*Decomposition*. Primary decomposer species groups were those which process significant amounts of dead organic matter (DOM) by direct consumption or through changing DOM structure to allow decomposition by other means (e.g., aeration and introduction of fungi and bacteria). These groups included ants^61^, isopods (Isopoda)^48^ and millipedes (Myriapoda: Diploploda)^48^. Secondary decomposers included species groups which are omnivorous but likely to have a lesser effect on decomposition rates. These included carabid and cerambycid beetles, craneflies (Diptera: Tipuloidea and Ptychopteridae) and harvestmen^66^,^67^.

*Carbon sequestration*. This functional group includes species which are capable of a net draw down and storage of CO_2_ from the atmosphere and storage in tissues. They include the photosynthesizing species groups of vascular plants (Tracheophyta), mosses (Bryophyta) and liverworts (Marchantiophyta)^68^,^69^. Note that, although per capita contributions of tree species may be much greater than smaller organisms such as mosses, net ecosystem functions depend on total abundance, total biomass and turnover of species, all of which can be higher for smaller organisms.

*Cultural value*. This category, rather than being based on biophysical functions leading to provisioning and regulating services, relates to cultural ecosystem services^70^. Here, we focused on a commonly used subset of cultural services comprising humans experiencing species in their natural environment^70^. We made the assumption that the rate of submission of biological records since 1970 in Great Britain generally reflects the relative value of different species groups with respect to the well-being benefits from experiencing them. We ranked species groups by the median number of records per species per decade submitted to British recording schemes and societies. We selected the top quartile of groups which included birds, butterflies, moths, vascular plants, bees and mammals. Although most biological records are submitted by a small proportion of the general public dedicated to natural history recording, these six groups do represent those that are most visible and popular with the general public (for example, regularly appearing in news reports). For example, searching for these six groups (common name in singular and plural) on Google gave rise to 3.49 billion hits in total, compared with 1.33 billion hits for all other 16 species groups combined. However, we prefer the former approach to identify cultural value because people are directly experiencing species before submitting their records. We conducted two analyses, one combining all these culturally important groups and one separating out culturally important animals from plants.

Note that the ecosystem service function categories described above are not mutually exclusive. In addition, particular species providing ecosystem functions may, in some cases, provide certain ecosystem disservices. For example, rabbits and deer provide enjoyment for people experiencing them in the natural environment, but can also cause damage to land at high abundances. These ecosystem disservices may need to be balanced with ecosystem functions provided by species on a case by case basis.

## Additional information

**How to cite this article:** Oliver, T. H. *et al.* Declining resilience of ecosystem functions under biodiversity loss. *Nat. Commun.* 6:10122 doi: 10.1038/ncomms10122 (2015).

## Supplementary Material

Supplementary Information Supplementary Figures 1-7, Supplementary Tables 1-3 and Supplementary References.

Supplementary Data 1 Names of all species included in the analysis, the group to which they belong and their categorisation for each ecosystem function as either primary (1), secondary (1*) or very limited- or non- function providers (0). Also provided are the trends in frequency of occurrence or abundance for each species (see main text).

## Figures and Tables

**Table 1:** Ecosystem functions provided by higher taxonomic groups in Great Britain.

				**Ecosystem function and service type**				
				**Provisioning**	**Provisioning**	**Regulating**	**Regulating**	**Cultural**
**Species group**	**Number of species analysed**	**Total number of occurrence records**	**Analysis year range**	**Pollination**	**Pest control**	**Decomposition**	**Carbon sequestration**	**Experiential value**
Ants	28	3,037	1970–2009	0	1	1	0	0
Bees	196	91,352	1970–2009	1	0	0	0	1
Birds	46	A*	1966–2011	0	1	0	0	1
Butterflies	59	A*	1976–2012	1	0	0	0	1
Carabid beetles	304	27,537	1970–2009	0	1	1*	0	0
Centipedes	30	1,251	1970–2009	0	1	0	0	0
Cerambycid beetles	31	417	1970–2009	1*	0	1*	0	0
Craneflies	67	1,208	1970–2009	0	0	1*	0	0
Dragonflies and damselflies	37	343,996	1970–2009	0	1	0	0	0
Crickets and earwigs	10	2,898	1970–2009	0	1*	0	0	0
Harvestmen	19	1,247	1970–2009	0	1	1*	0	0
Hoverflies	206	207,053	1970–2009	1	1	0	0	0
Isopods	27	3,781	1970–2009	0	0	1	0	0
Ladybird beetles	30	14,016	1970–2009	0	1	0	0	0
Mammals	30	A*	1984–2009	0	1	0	0	1
Millipedes	40	2,316	1970–2009	0	0	1	0	0
Mosses and liverworts	251	30,397	1970–2009	0	0	0	1	0
Moths	259	A*	1968–2007	1	0	0	0	1
Soldier beetles and glowworms	43	2,080	1970–2009	1*	1	0	0	0
Spiders	502	92,788	1970–2009	0	1	0	0	0
Vascular plants	2,025	683,261	1970–2009	0	0	0	1	1
Wasps	184	38,181	1970–2009	1*	1	0	0	0

Taxonomic groups correspond to national recording schemes or societies http://www.brc.ac.uk/recording-schemes). Scores of ‘1' indicate species in a group are primary ecosystem function providers; scores of ‘1*' indicate species in a group are secondary ecosystem function providers. Shown also are the total number of species and occurrence records analysed in each group after controls for recording effort. A* indicates that abundance rather than occurrence data were analysed.

## References

[b1] Mace G. M., Norris K. & Fitter A. H. Biodiversity and ecosystem services: a multilayered relationship. Trends Ecol. Evol. 27, 19–26 (2012). 21943703 10.1016/j.tree.2011.08.006

[b2] Balvanera P. *et al.* Quantifying the evidence for biodiversity effects on ecosystem functioning and services. Ecol. Lett. 9, 1146–1156 (2006). 16972878 10.1111/j.1461-0248.2006.00963.x

[b3] Cardinale B. J. *et al.* Biodiversity loss and its impact on humanity. Nature 486, 59–67 (2012). 22678280 10.1038/nature11148

[b4] Loreau M. & de Mazancourt C. Biodiversity and ecosystem stability: a synthesis of underlying mechanisms. Ecol. Lett. 16, 106–115 (2013). 23346947 10.1111/ele.12073

[b5] Oliver T. H. *et al.* Biodiversity and resilience of ecosystem functions. Trends Ecol. Evol 30, 673–684 (2015). 26437633 10.1016/j.tree.2015.08.009

[b6] Dirzo R. *et al.* Defaunation in the Anthropocene. Science 345, 401–406 (2014). 25061202 10.1126/science.1251817

[b7] Díaz S. *et al.* Functional traits, the phylogeny of function, and ecosystem service vulnerability. Ecol. and Evol. 3, 2958–2975 (2013). 10.1002/ece3.601 PMC3790543 24101986

[b8] Isaac N. J. B., van Strien A. J., August T. A., de Zeeuw M. P. & Roy D. B. Statistics for citizen science: extracting signals of change from noisy ecological data. Meth. Ecol. Evol. 5, 1052–1060 (2014).

[b9] Yachi S. & Loreau M. Biodiversity and ecosystem productivity in a fluctuating environment: the insurance hypothesis. Proc. Natl Acad. Sci. USA 96, 1463–1468 (1999). 9990046 10.1073/pnas.96.4.1463 PMC15485

[b10] Allan E. *et al.* More diverse plant communities have higher functioning over time due to turnover in complementary dominant species. Proc. Natl Acad. Sci. USA 108, 17034–17039 (2011). 21949392 10.1073/pnas.1104015108 PMC3193239

[b11] Hooper D. U. & Vitousek P. M. The effects of plant composition and diversity on ecosystem processes. Science 277, 1302–1305 (1997).

[b12] Cardinale B. J. *et al.* Effects of biodiversity on the functioning of trophic groups and ecosystems. Nature 443, 989–992 (2006). 17066035 10.1038/nature05202

[b13] Sih A., Englund G. & Wooster D. Emergent impacts of multiple predators on prey. Trends Ecol. Evol. 13, 350–355 (1998). 21238339 10.1016/s0169-5347(98)01437-2

[b14] Klanderud K. & Totland Ø. Simulated climate change altered dominance hierarchies and diversity of an alpine biodiversity hotspot. Ecology 86, 2047–2054 (2005).

[b15] Walker M. D. *et al.* Plant community responses to experimental warming across the tundra biome. Proc. Natl Acad. Sci. USA 103, 1342–1346 (2006). 16428292 10.1073/pnas.0503198103 PMC1360515

[b16] Straub C. S., Finke D. L. & Snyder W. E. Are the conservation of natural enemy biodiversity and biological control compatible goals? Biol. Control 45, 225–237 (2008).

[b17] Kattge J. *et al.* TRY—a global database of plant traits. Glob. Ch. Biol. 17, 2905–2935 (2011).

[b18] Luck G. W., Lavorel S., McIntyre S. & Lumb K. Improving the application of vertebrate trait-based frameworks to the study of ecosystem services. J. Anim. Ecol. 81, 1065–1076 (2012). 22435774 10.1111/j.1365-2656.2012.01974.x

[b19] Vandewalle M. *et al.* Functional traits as indicators of biodiversity response to land use changes across ecosystems and organisms. Biodivers. Conserv. 19, 2921–2947 (2010).

[b20] Winfree R., Fox J. W., Williams N. M., Reilly J. R. & Cariveau D. P. Abundance of common species, not species richness, drives delivery of a real-world ecosystem service. Ecol. Lett. 18, 626–635 (2015). 25959973 10.1111/ele.12424

[b21] Kleijn D. *et al.* Delivery of crop pollination services is an insufficient argument for wild pollinator conservation. Nat. Commun. 6, 7414 (2015). 26079893 10.1038/ncomms8414 PMC4490361

[b22] Woodcock B. A. *et al.* National patterns of functional diversity and redundancy in predatory ground beetles and bees associated with key UK arable crops. J. Appl. Ecol. 51, 142–151 (2014).

[b23] Cai W. *et al.* Increasing frequency of extreme El Nino events due to greenhouse warming. Nat. Clim. Chang. 4, 111–116 (2014).

[b24] Bommarco R., Lundin O., Smith H. G. & Rundlöf M. Drastic historic shifts in bumble-bee community composition in Sweden. Proc. R. Soc. Lond. B 282, 1812 (2011). 10.1098/rspb.2011.0647 PMC3223670 21676979

[b25] Lovell R., Wheeler B. W., Higgins S. L., Irvine K. N. & Depledge M. H. A systematic review of the health and well-being benefits of biodiverse environments. J. Toxicol. Environ. Health B Crit. Rev. 17, 1–20 (2014). 24597907 10.1080/10937404.2013.856361

[b26] Standish R. J. *et al.* Resilience in ecology: Abstraction, distraction, or where the action is? Biol. Cons. 177, 43–51 (2014).

[b27] Bullock J. M., Aronson J., Newton A. C., Pywell R. F. & Rey-Benayas J. M. Restoration of ecosystem services and biodiversity: conflicts and opportunities. Trends Ecol. Evol. 26, 541–549 (2011). 21782273 10.1016/j.tree.2011.06.011

[b28] Woodcock B. A. *et al.* Identifying time lags in the restoration of grassland butterfly communities: A multi-site assessment. Biol. Cons. 155, 50–58 (2012).

[b29] Roy D. B., Rothery P. & Brereton T. Reduced-effort schemes for monitoring butterfly populations. J. Appl. Ecol. 44, 993–1000 (2007).

[b30] Botham M. S., Brereton T. M., Middlebrook I., Randle Z. & Roy D. B. United Kingdom Butterfly Monitoring Scheme Report for 2012 Centre for Ecology and Hydrology (2013).

[b31] Fox R. *et al.* The State of Britain's Larger Moths Butterfly Conservation and Rothamsted Research (2013).

[b32] Freeman S. N., Noble D. G., Newson S. E. & Baillie S. R. Modelling population changes using data from different surveys: the Common Birds Census and the Breeding Bird Survey. Bird Study 54, 61–72 (2007).

[b33] Tracking Mammals Partnership. UK Mammals Update 2009, http://jncc.defra.gov.uk/page-5497 (2009).

[b34] Roy H. E. *et al.* Invasive alien predator causes rapid declines of native European ladybirds. Divers. Dist 18, 717–725 (2012).

[b35] Isaac N. J. B., van Strien A. J., August T. A., de Zeeuw M. P. & Roy D. B. Extracting robust trends in species' distributions from unstructured opportunistic data: a comparison of methods. BioRXiv http://dx.doi.org/10.1101/006999 (2014).

[b36] Norris K. J. *et al.* Chapter 4: Biodiversity in the context of ecosystem services. in The UK National Ecosystem Asssessment Technical Report UNEP-WCMC (2011).

[b37] Straub C. S. & Snyder W. E. Species Identity Dominates the Relationship between Predator Biodiversity and Herbivore Suppression. Ecology 87, 277–282 (2006). 16637351 10.1890/05-0599

[b38] UK Ladybird Survey, http://www.ladybird-survey.org/species_list.aspx (2014).

[b39] Beetles and Beetle Recording in Great Britain, http://www.coleoptera.org.uk/home (2014).

[b40] Gibson R. L., Elliott N. C. & Schaefer P. Life History and Development of Scymnus frontalis (Fabricius) (Coleoptera: Coccinellidae) on Four Species of Aphid. J. Kans. Entomol. Soc. 65, 410–415 (1992).

[b41] Lu W. & Montgomery M. E. Oviposition, Development, and Feeding of *Scymnus (Neopullus ) sinuanodulus* (Coleoptera: Coccinellidae): A Predator of *Adelges tsugae* (Homoptera: Adelgidae). Ann. Entomol. Soc. Am. 94, 64–70 (2001).

[b42] Biddinger D. J., Weber D. C. & Hull L. A. Coccinellidae as predators of mites: Stethorini in biological control. Biological Control 51, 268–283 (2009).

[b43] Sujii E. R. *et al.* Community of natural enemies and natural biological control of the aphid *Aphis gossypii* Glover (Hemiptera: Aphididae) and cotton leafworm *Alabama argillacea* Hübner (Lepidoptera: Noctuidae) in the cotton crop. Journal Arquivos do Instituto Biológico (São Paulo) 74, 329–336 (2007).

[b44] Orthoptera and Allied Insects Recording Scheme http://www.orthoptera.org.uk/ (2014).

[b45] Marshall J. A., Haes E. C. M. & Ovended D. Grasshoppers and Allied Insects of Great Britain and Ireland Harley Books (1988).

[b46] The Royal Society for the Protection of Birds (RSPB). Bird guide. https://www.rspb.org.uk/discoverandenjoynature/discoverandlearn/birdguide/ (2014).

[b47] Hill D. S. Agricultural insect pests of temperate regions and their control Cambridge University Press (1987).

[b48] Snyder B. A. & Hendrix P. F. Current and Potential Roles of Soil Macroinvertebrates (Earthworms, Millipedes, and Isopods) in Ecological Restoration. Restoration Ecology 16, 629–636 (2008).

[b49] Forup M. L. & Memmott J. The Restoration of Plant–Pollinator Interactions in Hay Meadows. Restoration Ecology 13, 265–274 (2005).

[b50] Jauker F., Bondarenko B., Becker H. C. & Steffan-Dewenter I. Pollination efficiency of wild bees and hoverflies provided to oilseed rape. Agricultural and Forest Entomology 14, 81–87 (2012).

[b51] Gess S. K. The pollen wasps: ecology and natural history of the Masarinae Harvard University Press (1996).

[b52] Symondson W. O. C., Sunderland K. D. & Greenstone M. H. Can generalist predators be effective biocontrol agents? Annu. Rev. Entomol. 47, 561–594 (2002). 11729085 10.1146/annurev.ento.47.091201.145240

[b53] Chiverton P. A. Predator density manipulation and its effects on populations of Rhopalosiphumpadi (Horn.: Aphididae) in spring barley. Ann. Appl. Biol. 109, 49–60 (1986).

[b54] Solomon M. G. *et al.* Biocontrol of Pests of Apples and Pears in Northern and Central Europe - 3. Predators. Biocontrol Science and Technology 10, 91–128 (2000).

[b55] Dixon A. F. G. Insect Predator-Prey Dynamics: Ladybird Beetles and Biological Control Cambridge University Press (2000).

[b56] Chiverton P. A. Predation of Rhopalosiphum padi (Homoptera: Aphididae) by polyphagous predatory arthropods during the aphids' pre-peak period in spring barley. Ann. Appl. Biol. 111, 257–269 (1987).

[b57] Mills N. J. & Getz W. M. Modelling the biological control of insect pests: a review of host-parasitoid models. Ecol. Mod. 92, 121–143 (1996).

[b58] Shaalan E. A. S. & Canyon D. V. Aquatic insect predators and mosquito control. Trop. Biomed. 26, 223–261 (2009). 20237438

[b59] Sergeeva T. K. Opiliones guild: Structure and trophic relations. Zool. Zhurnal 78, 1172–1178 (1999).

[b60] Gomez-Polo P. *et al.* Identification of the most common predatory hoverflies of Mediterranean vegetable crops and their parasitism using multiplex PCR. J. Pest. Sci. 87, 371–378 (2014).

[b61] Del Toro I., Ribbons R. R. & Pelini S. L. The little things that run the world revisited: a review of ant-mediated ecosystem services and disservices (Hymenoptera: Formicidae). Myrmecol. News 17, 133–146 (2012).

[b62] Styrsky J. D. & Eubanks M. D. Ecological consequences of interactions between ants and honeydew-producing insects. Proc. Roy. Soc. B 274, 151–164 (2007). 10.1098/rspb.2006.3701 PMC1685857 17148245

[b63] Kirk D. A., Evenden M. D. & Mineau P. in Current Ornithology eds Nolan V., Ketterson E. D. Ch. 5, 175–269Springer (1996).

[b64] Churchfield S., Hollier J. & Brown V. K. The effects of small mammal predators on grassland invertebrates, investigated by field exclosure experiment. Oikos 60, 283–290 (1991).

[b65] Boyles J. G., Cryan P. M., McCracken G. F. & Kunz T. H. Economic importance of bats in agriculture. Science 332, 41–42 (2011). 21454775 10.1126/science.1201366

[b66] Perel T. S., Karpache L. O. & Yegorova E. V. The role of Tipulidae (Diptera) larvae in decomposition of forest litter-fall. Pedobiologia 11, 66–70 (1971).

[b67] Foit J. Distribution of early-arriving saproxylic beetles on standing dead Scots pine trees. Agric. For. Entomol. 12, 133–141 (2010).

[b68] Chapin F. S., Schulze E. & Mooney H. A. The Ecology and Economics of Storage in Plants. Ann. Rev. Ecol. Syst. 21, 423–447 (1990).

[b69] De Deyn G. B., Cornelissen J. H. C. & Bardgett R. D. Plant functional traits and soil carbon sequestration in contrasting biomes. Ecol. Lett. 11, 516–531 (2008). 18279352 10.1111/j.1461-0248.2008.01164.x

[b70] Church A. *et al.* Chapter 16: Cultural Services in The UK National Ecosystem Asssessment Technical Report http://uknea.unep-wcmc.org/LinkClick.aspx?fileticket=m%2BvhAV3c9uk%3D&tabid=82UK National Ecosystem Assessment, UNEP-WCMC (2011).

