# Supplementary material for: Declining resilience of ecosystem functions under biodiversity loss
Source: Nat Commun. 2015 Dec 8;6:10122. doi: 10.1038/ncomms10122 (PMC4686828; doi:10.1038/ncomms10122)
Supplement: Supplementary Information — Supplementary Figures 1-7, Supplementary Tables 1-3 and Supplementary References. [file ncomms10122-s1.pdf]

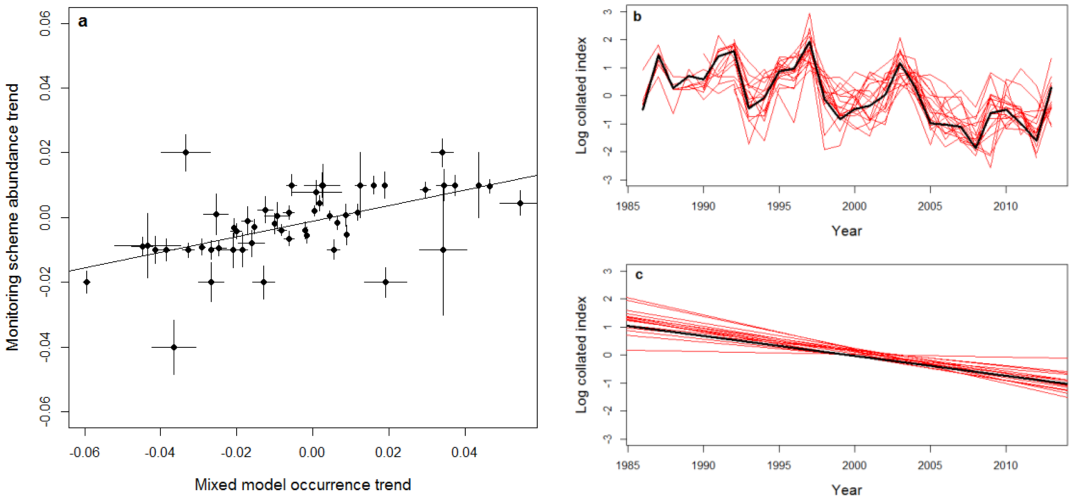

Supplementary Figure 1, Panel a: relationship between linear trends in butterfly species occurrence from mixed models fitted to distribution data versus abundance trends from monitoring transects (Type II major axis regression: n = 53, R^2 = 0.31, p<0.01). Error bars represent standard errors of linear trend coefficients. Note that there may be both ecological and sampling methodology reasons for imperfect relationships, but trends derived from the two data types show strong correlations suggesting that they capture inter-related aspects of abundance and frequency of occurrence change. Panels b and c show log collated abundance indices for an example species, *Aglais urticae*, chosen because it is particularly widespread thereby allowing individual analysis for the greatest number of counties (GB sub-regions). The national average annual abundance (panel b, solid black line) and the linear trend over time (panel c, solid black line) reasonably reflect changes across most counties (red lines in both panels). Log collated annual abundance indices were calculated following methods of [1]. Abundance trends (panels a and c) were calculated from linear regression of these annual collated indices against year. For panels b and c, minimum data requirements to fit indices for individual counties were 20 years of recording data from 1985 with at least five sites recorded in each year.

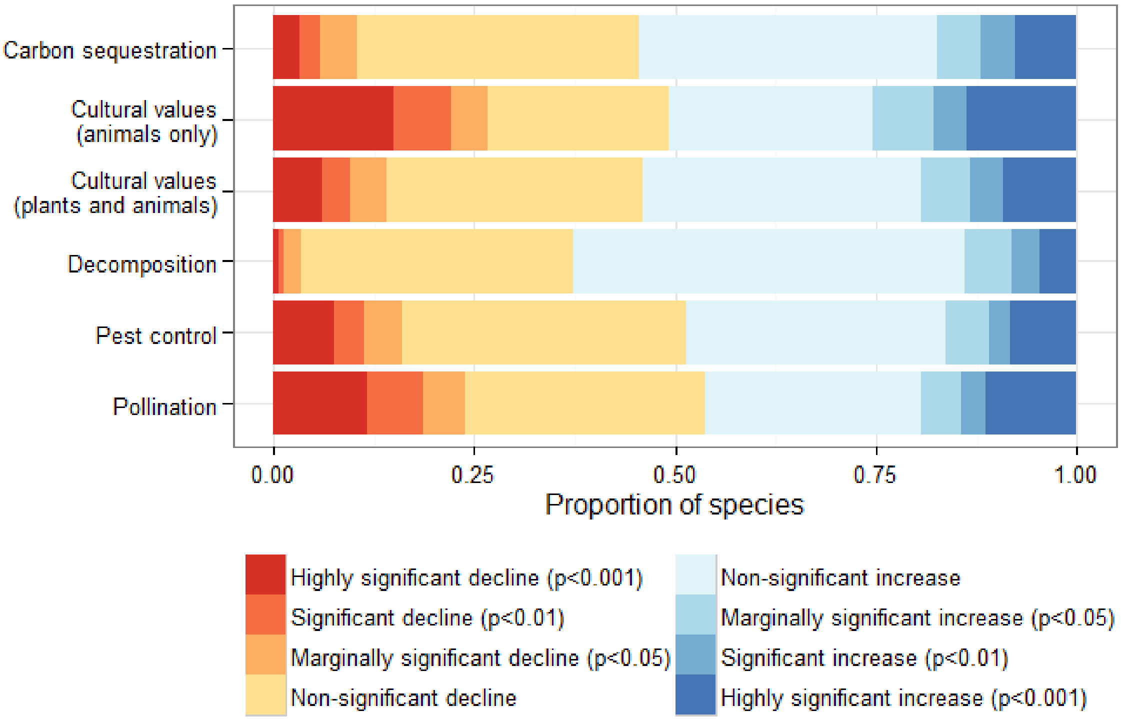

Supplementary Figure 2, Proportion of species in different functional groupings showing significant changes in frequency of occurrence in Great Britain between 1970 and 2010. Here, functional groupings include also species groups categorised as ‘secondary’ in providing particular ecosystem functions. Total sample sizes for respective rows are as follows: n = 2276, 590, 2615, 516, 1447, 978.

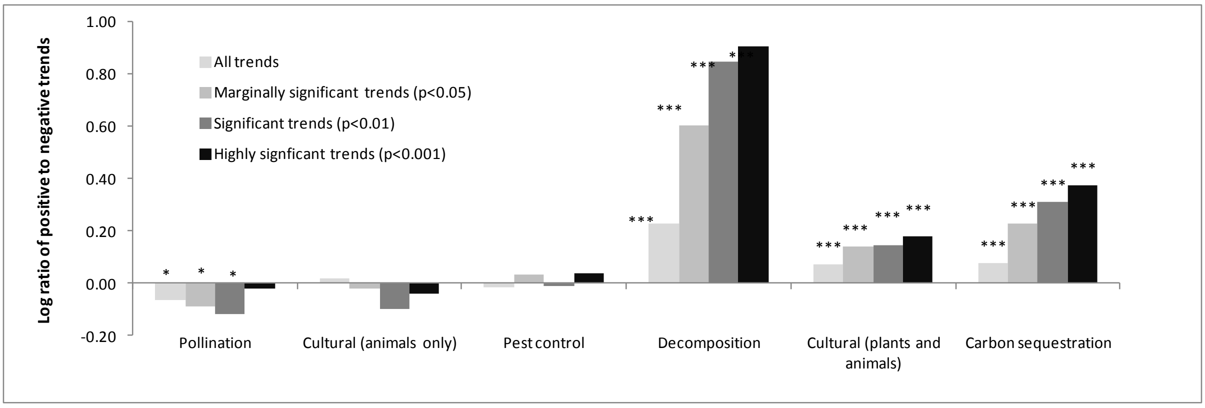

Supplementary Figure 3, Log ratio of numbers of increasing versus decreasing species in different functional groups. Here, functional groupings include species groups also categorised as ‘secondary’ in providing particular ecosystem functions. The different bars indicate different significance levels for individual species trends. A positive ratio indicates more species in a given functional group are increasing. Differences in the balance of increasing versus decreasing species is assessed using a proportion test (* p<0.05; *** p<0.001).

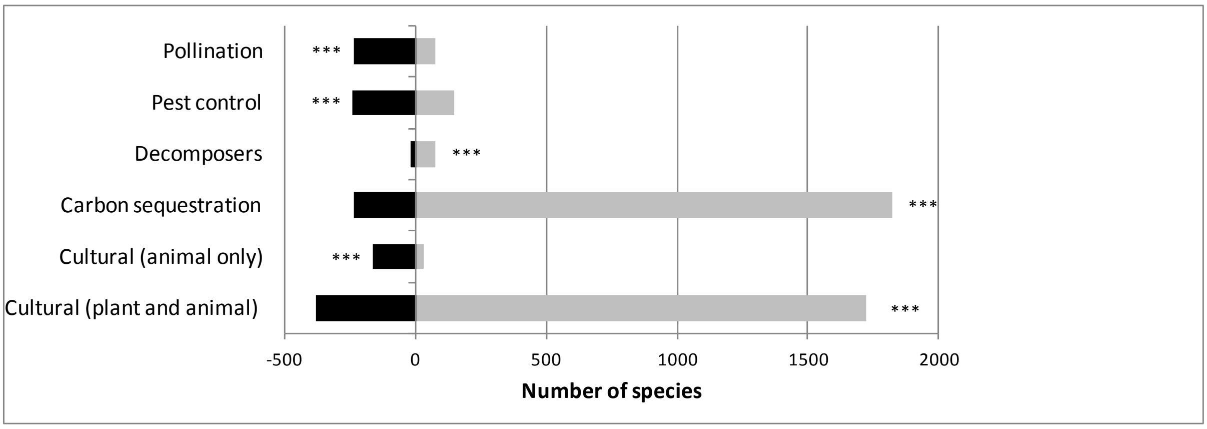

Supplementary Figure 4, Numbers of species showing declines in frequency of occurrence (at p<0.05; dark grey bars) versus the number of new species arriving in Great Britain since 1970 (light grey bars). Here, functional groupings also include species groups categorised as ‘secondary’ in providing particular ecosystem functions. Asterisks indicate significantly different proportions (*** p<0.001).

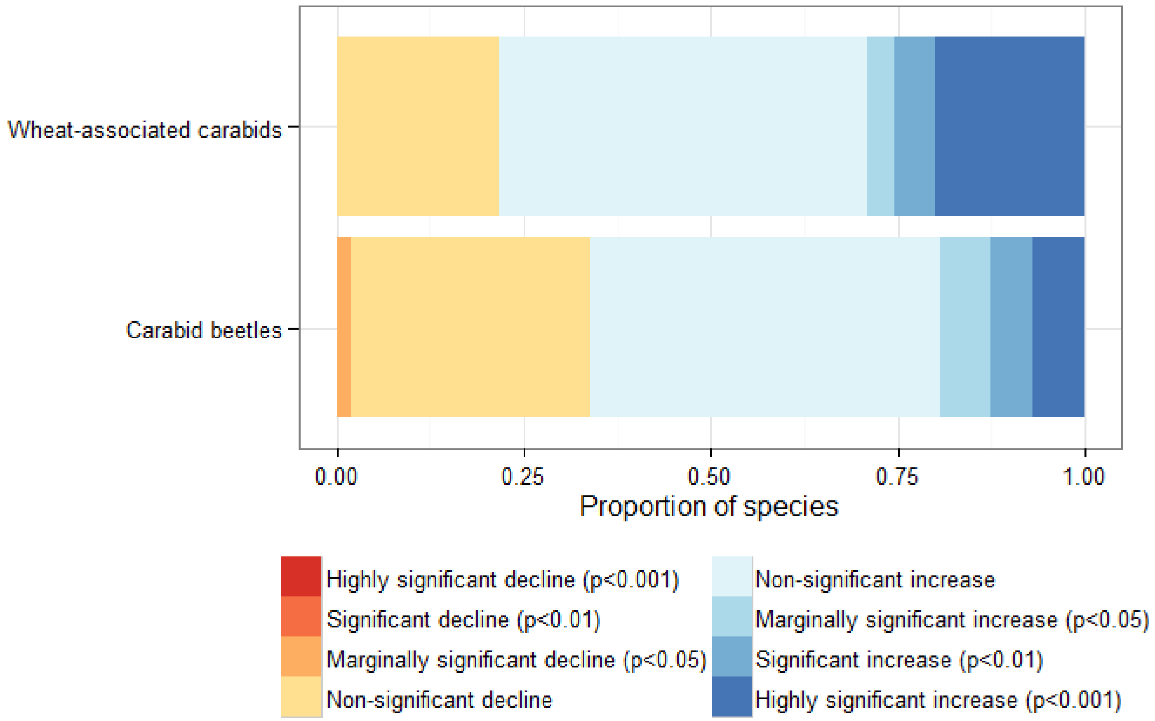

Supplementary Figure 5, Proportion of species showing significant changes in frequency of occurrence in Great Britain between 1970 and 2010 for carabid beetles strongly associated with wheat (n = 55) versus all carabid beetles with data available (n = 304). There was no significant difference in the overall proportion of species with any positive trends (p = 0.11), those with significant increases (with > 95% confidence threshold on individual trend significance; p = 0.15), or those with significant declines (p = 0.61).

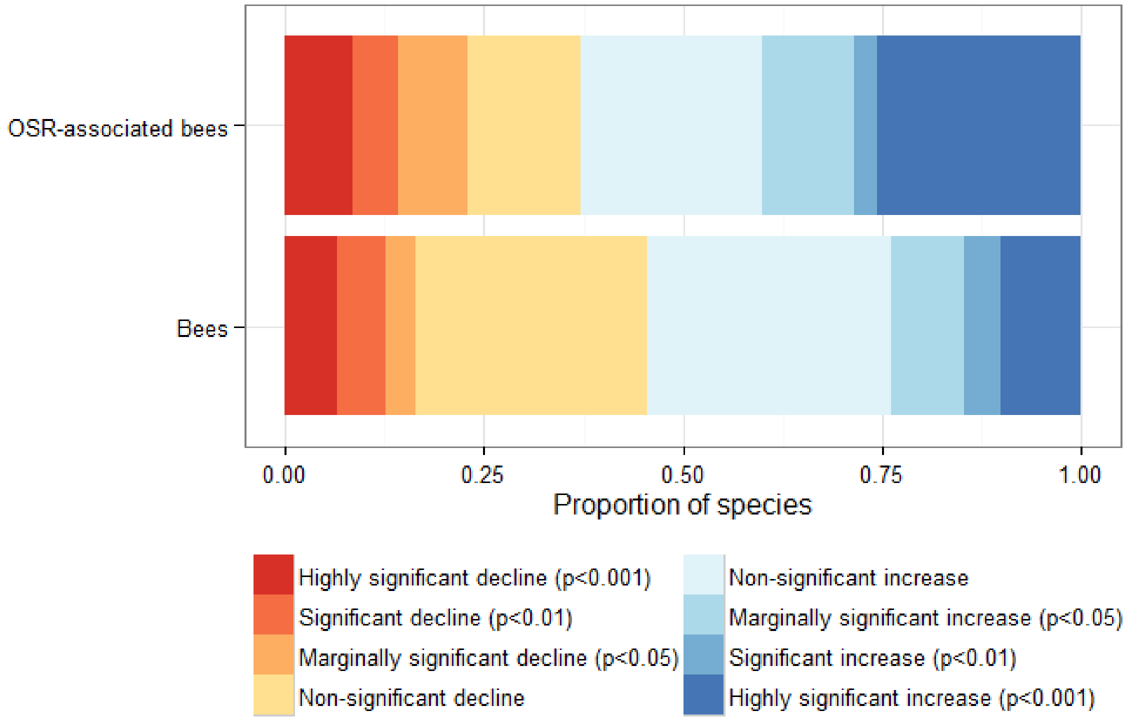

Supplementary Figure 6, Proportion of species showing significant changes in frequency of occurrence in Great Britain between 1970 and 2010 for bees strongly associated with oil seed rape (OSR) (n = 35) versus all bees with data available (n = 196). There was no significant difference in the overall proportion of species with any positive trends (p = 0.47), those with significant increases (with a 95% confidence threshold on individual trend significance; p = 0.08), or those with significant decreases (p = 0.49).

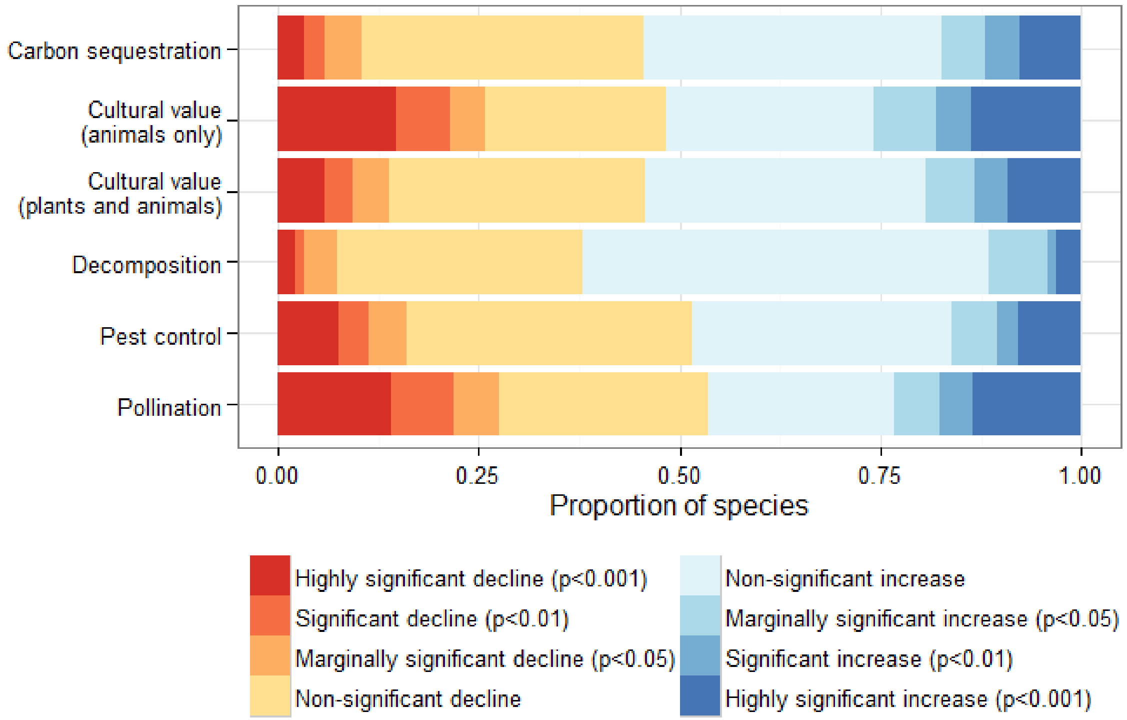

Supplementary Figure 7, Repeat of the analysis of primary ecosystem function-providing species groups (i.e. Figure 1), but excluding 81 species (comprising moths, butterflies, carabid and cerambycid beetles) which are crop pests in temperate regions.

Supplementary Table 1, Ecosystem functions studied with their categorisation under the UK National Ecosystem Assessment (UKNEA) conceptual framework and The Common International Classification of Ecosystem Services (CICES V4.3). The rows show broad terrestrial species groups that are of ‘high importance’ for each function, as identified by the UK NEA (summary Table 4.2[2]; shown here as dark shaded boxes). The bold bounding box includes species groups included in this study. Bryophytes are not identified of importance for climate regulation in the UK NEA summary table, but this may be an error. In the UKNEA text, peatlands (dominated by *Sphagnum* bryophyte species) are cited as being of ‘exceptional importance for carbon sequestration’[3], and so they are highlighted in light grey box here to indicate this discrepancy

**Ecosystem function**	Pollination	Pest control	Decomposition	Carbon Sequestration	Experiential value
**UKNEA final ecosystem service**	Pollination	Disease and pest regulation	Waste breakdown & detoxification	Climate regulation	No equivalent category
**CICES Class**	Pollination and seed dispersal	Pest control	Bio-remediation by micro-organisms, algae, plants, and animals & Decomposition and fixing processes	Global climate regulation by reduction of greenhouse gas concentrations & Micro and regional climate regulation	Experiential use of plants, animals and land-/seascapes in different environmental settings
**Species group**					
Lower plants (bryopytes)					
Higher plants					
Invertebrates					
Birds					
Mammals					
Microorganisms					
Fungi					
Fish					
Amphibians					
Reptiles					

Supplementary Table 2, Percentage of increasing versus decreasing species grouped by ecosystem function. Here, functional groupings include species groups categorised as ‘primary’ in providing particular ecosystem functions. Significant differences were assessed using a binomial test (all trends) or proportion test (for significant trends; * p<0.05; *** p<0.001). Total sample sizes for respective rows are as follows: n = 2276, 590, 2615, 95, 1447, 720.

Significance level for individual trends:	All trends				p<0.05				p<0.01				p<0.001			
	% declining	% increasing	Log ratio		% declining	% increasing	Log ratio		% declining	% increasing	Log ratio		% declining	% increasing	Log ratio	
Carbon sequestration	45.6	54.4	0.08	***	10.3	17.3	0.23	***	5.8	11.9	0.31	***	3.3	7.7	0.37	***
Cultural services (animals only)	49.0	51.0	0.02	NS	26.6	25.4	-0.02	NS	22.2	17.8	-0.10	NS	15.0	13.7	-0.04	NS
Cultural services (plants and animals)	46.0	54.0	0.07	***	14.1	19.4	0.14	***	9.6	13.2	0.14	***	6.0	9.1	0.18	***
Decomposition	37.9	62.1	0.21	*	7.4	11.5	0.19	NS	3.2	4.2	0.12	NS	2.1	3.2	0.19	NS
Pest control	51.0	49.0	-0.02	NS	16.0	16.9	0.02	NS	11.3	10.9	-0.02	NS	7.6	8.0	0.02	NS
Pollination	54.0	46.0	-0.07	*	27.9	23.2	-0.08	*	22.5	17.6	-0.11	*	14.4	13.6	-0.02	NS

Supplementary Table 3, Proportion of increasing versus decreasing species grouped by ecosystem function. Here, functional groupings also include species groups categorised as ‘secondary’ in providing particular ecosystem functions. Significant differences were assessed using a binomial test (all trends) or proportion test (for significant trends; * p<0.05; *** p<0.001). Total sample sizes for respective rows are as follows: n = 2276, 590, 2615, 516, 1447, 978.

**ES2**																
Significance level for individual trends:	All trends				p<0.05				p<0.01				p<0.001			
	% declining	% increasing	Log ratio		% declining	% increasing	Log ratio		% declining	% increasing	Log ratio		% declining	% increasing	Log ratio	
Carbon sequestration	45.6	54.4	0.08	***	10.3	17.3	0.23	***	5.84	11.91	0.31	***	3.3	7.7	0.37	***
Cultural services (animals only)	48.9	51.1	0.02	NS	26.5	25.4	-0.02	NS	22.12	17.73	-0.10	NS	15.0	13.7	-0.04	NS
Cultural services (plants and animals)	45.8	54.2	0.07	***	14.1	19.4	0.14	***	9.56	13.30	0.14	***	6.0	9.1	0.18	***
Decomposition	37.2	62.8	0.23	***	3.5	14.0	0.60	***	1.16	8.14	0.85	***	0.6	4.7	0.90	***
Pest control	50.8	49.2	-0.01	NS	15.8	17.0	0.03	NS	11.17	10.97	-0.01	NS	7.4	8.1	0.04	NS
Pollination	53.7	46.3	-0.06	*	23.7	19.4	-0.09	*	18.66	14.30	-0.12	*	11.8	11.3	-0.02	NS

Supplementary references

1 Roy, D. B., Rothery, P. & Brereton, T. Reduced-effort schemes for monitoring butterfly populations. *J. Appl. Ecol.* **44**, 993-1000 (2007).

2 Norris, K. J. & et al. *Chapter 4: Biodiversity in the context of ecosystem services* in *The UK National Ecosystem Asssessment Technical Report* (UNEP-WCMC, 2011).

3 Church, A. & et al. *Chapter 16: Cultural Services* in *The UK National Ecosystem Asssessment Technical Report* (http://uknea.unep-wcmc.org/LinkClick.aspx?fileticket=m%2BvhAV3c9uk%3D&tabid=82 UK National Ecosystem Assessment, UNEP-WCMC, Cambridge, UK, 2011).
